# Effects of Background Music on Attentional Networks of Children With and Without Attention Deficit/Hyperactivity Disorder: Case Control Experimental Study

**DOI:** 10.2196/53869

**Published:** 2024-07-18

**Authors:** Camila Guimarães Mendes, Jonas Jardim de Paula, Débora Marques Miranda

**Affiliations:** 1 Department of Pediatrics Federal University of Minas Gerais Belo Horizonte Brazil; 2 Department of Mental Health Federal University of Minas Gerais Belo Horizonte Brazil

**Keywords:** attention, background music, ADHD, children, adolescents, music, attention network, effects, preliminary study, attention deficit/hyperactivity disorder

## Abstract

**Background:**

To sustain performance during a task that requires attention may be a challenge for children with attention deficit/hyperactivity disorder (ADHD), which strongly influences motivation for tasks and has been connected to the level of arousal.

**Objective:**

This study aimed to analyze the effect of musical stimulus on attentional performance in children with ADHD and typically developing children.

**Methods:**

A total of 76 boys (34 with ADHD and 42 typically developing) performed the Attention Network Test (ANT) for children under 2 experimental conditions (with and without music). Four attentional measures were extracted from the ANT. We tested the effect of the experimental condition and its interaction with the group using repeated measures ANOVA.

**Results:**

We found no significant main effects or interactions for the reaction times of the alerting, orienting, and conflict attentional networks of the ANT (all *P*>.05). Regarding ANT errors, we found a significant main effect for music, with a moderate effect size (*F*_1,72=_9.83; *P*=.03; ηp^2^=0.06) but the condition×group interaction was not significant (*F*_1,72_=1.79; *P*=.18). Participants made fewer errors when listening to music compared to the control condition.

**Conclusions:**

Music seems not to interfere in the attentional network in children and adolescents. Perhaps background music affects motivation. Future studies will be needed to validate this.

**Trial Registration:**

ReBEC.gov U1111-12589039; https://ensaiosclinicos.gov.br/rg/RBR-8s22sh8

## Introduction

Attention deficit/hyperactivity disorder (ADHD) is a common neurodevelopmental disorder characterized by harmful levels of inattention, impulsivity, and hyperactivity [1]. ADHD exhibits considerable heterogeneity, with individuals’ symptoms reflecting impairments in different cognitive aspects [2], causing distress or problems at home, at school, and with peers [3]. Impaired cognitive aspects in ADHD include frequent compromises in executive functions (ie, working memory, inhibitory control, cognitive flexibility, planning, and problem-solving), self-regulation states (ie, the purposeful or automatic mechanisms that enable behavior to be adapted appropriately to a changing context), motivation (ie, temporal reward discounting), and time perception (ie, the ability to discriminate and compare time intervals) [4-6]. The hyperactivity and inattention levels of children with ADHD are noticeably higher than expected.

The attentional modeling of Berger and Posner [7] provides an appropriate theoretical framework to account for ADHD dysfunction because it conceptualizes most of the abilities mentioned above as part of attentional networks, such as alerting (ie, arousal of the cognitive system), orienting (ie, allocating attentional focus in the visual field), and executive control (ie, ability to control our own behavior, resolve conflict, and inhibit impulsive responses). A task that requires extra effort for children to sustain performance may be a challenge for children with ADHD, especially in suboptimal conditions [8]. On the other hand, effort is determined by the motivation to perform the task and has been connected to the level of arousal and activation [8,9]. This explains why children with ADHD, who are easily distracted by external stimuli, may benefit from stimuli that promote increased alertness and consequently improve performance in the task [10,11].

A recent systematic review showed listening to music without lyrics that was chosen by the listener seemed to improve performance in tasks requiring attention [12]. Music enhances arousal, can affect mood, and increases motivation, especially when it is preferred by the listener, potentially benefiting the learning process through emotional processes [13-15]. This heightened state of alertness and pleasant mood can enhance attentional resources, allowing the listener to concentrate better and sustain focus on cognitive tasks [16,17]. Music holds the potential to augment the emotion regulation abilities and mood of young individuals in their daily experiences [18].

Knowledge of the effect of music on the cognitive function of individuals with ADHD is still limited due to inconsistent results [19]. Among studies that evaluated music as a form of stimulation in ADHD, 2 reported improvements in mathematical problem-solving [20,21], while another study assessing schoolwork completion (including math, reading, reading comprehension, and language arts) showed no significant difference in cognitive function. The heterogeneity in the methodology of these studies makes it difficult to draw conclusions on the true effect of music on task performance. Nevertheless, a recent review indicated that listening to music can reduce symptoms of ADHD and improve timing perception and regulation [22], which are important for the functionality and well-being of this population.

At present, there is a lack of data assessing the impact of music listening on the attention networks of children with ADHD. Therefore, the aim of this study was to investigate the effects of music listening on the attention networks—namely, alerting, orienting, and conflict—in children with ADHD and typically developing children, while also exploring the relationship with the attentional profile of these children. Given that previous studies involving ADHD [23,24] incorporated measures of error types alongside conventional assessments of the 3 attention networks, we will also examine whether music influences error rates during task performance. Our hypothesis is that music may enhance attentional performance in children with ADHD differently from their typically developing peers.

## Methods

### Study Design

This preliminary, experimental, repeated-measures study was conducted from 2019 to 2022 to explore the impact of music listening compared to no music listening on attention performance. We enrolled boys aged 10 to 12 years, both with and without ADHD, who completed the Attention Network Test (ANT) for children twice under randomized conditions.

### Recruitment

**Figure 1:**
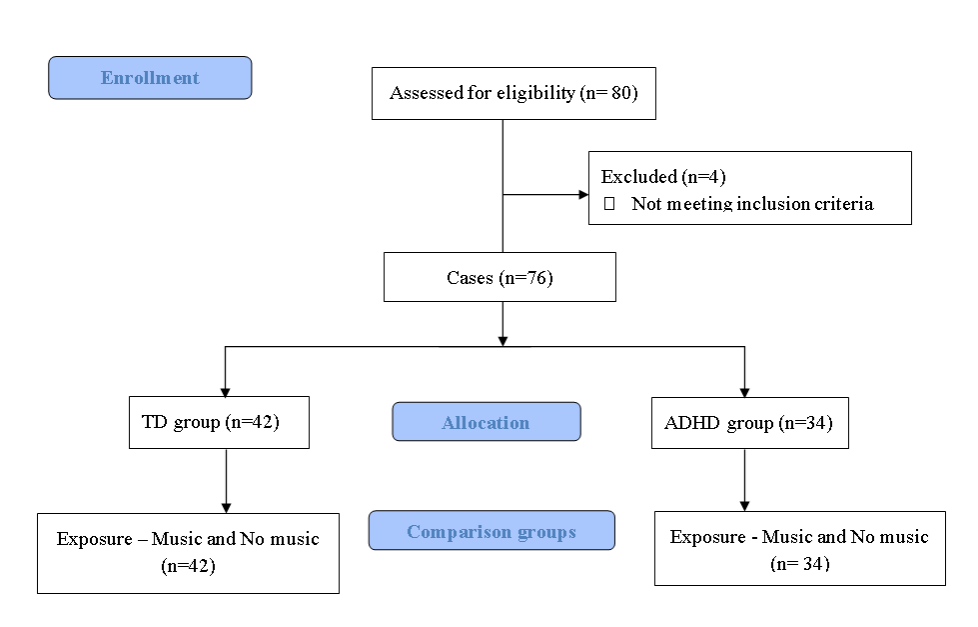
Flow diagram of the research process. ADHD: attention deficit/hyperactivity disorder; TD: typically developing.

A total of 76 boys aged 10 to 12 years participated, comprising 34 with ADHD and 42 without ADHD (Figure 1). This age range was selected based on evidence indicating that children younger than 10 years are still developing their musical preferences, while adolescents tend to be more receptive to unfamiliar music styles [25]. Given that the musical stimulus in our study needed to be familiar and preferred by the listener, we focused on the age range of 10 to 12 years. Also, only boys were included, because the majority of children treated at the university hospital were male.

Participants were recruited from a university hospital that provides psychiatric care for children and adolescents with ADHD, as well as pediatric follow-up for healthy individuals. Children with ADHD met the *Diagnostic and Statistical Manual of Mental Disorders, Fifth Edition*, criteria [1] and underwent assessment using the semistructured Schedule for Affective Disorders and Schizophrenia for School-Age Children–Present and Lifetime (K-SADS-PL) [26]. Moreover, they achieved scores at or above the 10th percentile on the Brazilian version of Raven’s Colored Progressive Matrices intelligence test [27].

The control group was selected from the local community between 2021 and 2022. This group was matched with the ADHD group in terms of age and socioeconomic status, and they met the inclusion criteria by not having a diagnosis of ADHD or by not scoring above the cutoff points on screening questionnaires for ADHD. These cutoff points included having more than 5 ADHD symptoms identified by the Swanson, Noland, and Pelham Scale IV (SNAP IV) or having a *t* score greater than 70 on the ADHD scale of the Child Behavior Checklist for Ages 6-18 (CBCL/6-18) [28,29].

### Intervention

The intervention required participants to perform an attention task in 2 different conditions: with music and without music. The music selection comprised 5 songs chosen through interviews with children aged 10 to 12 years, who shared their favorite and most frequently listened-to songs. These songs were played during the test. It is important to note that the children interviewed about their favorite music were not necessarily participants in the study.

To gauge the emotional connection between listeners and songs (including familiarity, preference, mood, and arousal), a questionnaire was administered. Participants listened to song excerpts and answered questions such as “Do you know this song?” (answers were yes, maybe, or no), “Do you like this song?” (answers were yes, neutral, or no), and “How do you feel listening to these songs?” using the adapted Self-Assessment Manikin Scale [30]. A 5-point Likert scale was used to rate subjective mood (1=very sad, 2=sad, 3=neutral, 4=happy, and 5=very happy) and arousal (1=nonarousal, 2=low arousal, 3=neutral, 4=arousal, and 5=high arousal) based on images pointed to by the children. This questionnaire was administered before the ANT to ensure that the results were not influenced by the child’s performance. It can be found in Multimedia Appendix 1.

To prevent experimenter bias, the order of play of the songs was determined through a random drawing using Microsoft Excel (Table 1). The music was played using a Samsung Galaxy J5 and Shure 440 Hz headphones, with the volume standardized to the same level for all participants.

**Table 1:** List of selected songs and their order of play.

Order	Title	Duration (min:s)
1	Fortnite OST–Battle Royale Menu Music (Rock Version) [31]^a^	3:50
2	Alone (Mashmallo) – Modified [32]^b^	3:19
3	Free Fire New EPIC Theme Song [33]^c^	3:56
4	Herobrine’s Life (Instrumental) [34]^d^	4:00
5	Olha a explosão (Mc Kevinho) – Modified [35]^b^	3:07

^a^This song is part of the game Fortnite and was formerly played in the Battle Royale menu and when a player wins the Battle Royale mode. It was composed by Rom Di Prisco; all content belongs to Epic Games.

^b^The original song was modified with Audacity (version 2.3.2; Audacity Team), an audio editor, to remove the voices.

^c^This song is the theme song of Free Fire 2019.

^d^This song is a Minecraft parody of the song “Something Just Like This” by The Chainsmokers and Coldplay.

### Procedure

**Figure 2:**
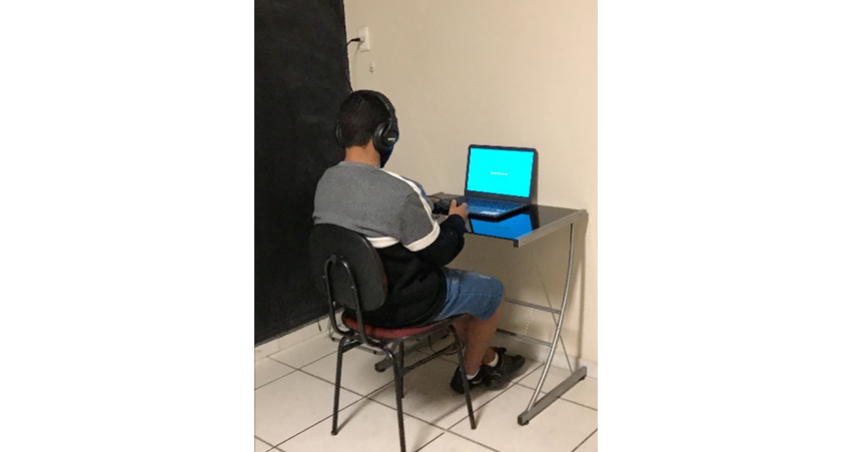
Experimental setup.

The attention task involved the child version of the ANT [36] under 2 conditions (with and without music). The child ANT was run using E-prime (version 2.0 professional; Psychology Software Tools) downloaded on a Samsung notebook from the webpage of Jin Fan [37]. All participants faced the laptop on the table in a comfortable, seated position (Figure 2). Prior to the ANT, the experimenter administered Conner’s Continuous Performance Test (CCPT) to the children. The entire procedure lasted approximately 1 hour and 30 minutes.

The first author (CGM) contacted parents or caregivers via telephone to arrange the experiment day. For the ADHD group, the experiment was scheduled on the same day as the participant’s psychiatrist appointment at the university hospital, or another agreed-upon day, to enhance adherence to the intervention. Children were individually escorted to a quiet office while their parents completed behavior scales and a sociodemographic questionnaire in the waiting room. It is crucial to highlight that this was a clinical sample. All children were under psychiatric monitoring and had a confirmed diagnosis of ADHD prior to participation.

For the control group, the experiment was scheduled on the most convenient day and location for caregivers, provided the child met all eligibility criteria. Screening for ADHD was conducted using the SNAP-IV and CBCL/6-18 scales, which were completed by the parents as a web form. When the experiment was held at a participant’s home, the child performed the task on a table in the quietest area of the house.

Additionally, all children and caregivers were asked to complete a semistructured questionnaire, which can be found in Multimedia Appendix 2, before starting the task.

### Measures

#### Primary Outcome Measure

**Figure 3:**
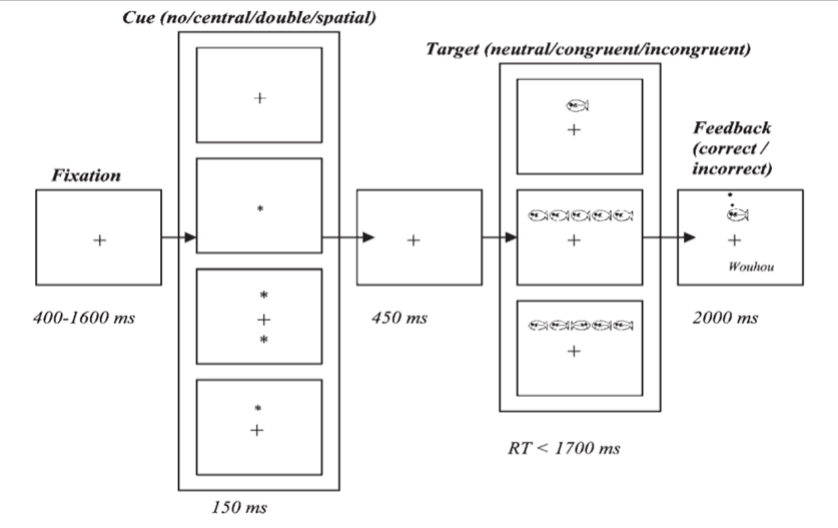
Schematic of the child version of the ANT. In the actual task, the background color for every display is blue and the fish appear in yellow; the auditory feedback was used only in practice trials. RT: reaction time.

The ANT (child version) was designed to assess 3 attention networks (alerting, orienting, and conflict) within a single task framework based on the model described by Posner and Petersen [38]. In this version, participants are instructed to feed a central colorful fish by pressing a joystick button corresponding to the direction (left or right) in which it swims. The fish may appear alone or accompanied by other fish moving in the same or opposite direction (neutral, congruent, or incongruent stimuli) combined with various cuing conditions (no cue, central cue, double cue, and spatial cue) [38] (Figure 3).

Originally, the task comprised 24 practice trials followed by 3 experimental blocks of 48 trials each. Since children completed the task twice (with and without music), practice rounds were administered separately. This procedure typically lasted approximately 45 minutes, including 5 minutes of practice and 15-minute rounds of 48 trials each, with 1-2 minute rest intervals. Psychometric properties of the ANT were assessed with a sample size of 40, yielding test-retest reliabilities of 0.52, 0.61, and 0.77 for the alerting, orienting, and conflict measures, respectively [39]. Additionally, with a sample size of 104, test-retest reliabilities of 0.36, 0.41, and 0.81 were reported for the alerting, orienting, and conflict measures, respectively [40].

#### Secondary Outcomes

The SNAP IV is a screening scale for ADHD and oppositional defiant disorder (ODD) based on the *Diagnostic and Statistical Manual of Mental Disorders, Fourth Edition* criteria [29]. It consists of 26 items divided into subsets of symptoms (inattention, hyperactivity/impulsivity, and ODD) rated on a 4-point Likert scale ranging from 0 (not at all) to 3 (very much). Scores can be computed using 3 methods: averaging scores for each dimension, summing total scores, or counting the number of symptoms [29]. We used the symptom count to screen for ADHD, while the second and third methods were used for sample characterization. In a Brazilian sample, parental assessment of the SNAP IV demonstrated robust psychometric properties, with Cronbach α values of 0.94 and 0.92 for the inattention and hyperactivity scales, respectively [29].

The CBCL/6-18 is a self-report questionnaire assessing behaviors with 118 items scored as 0 (not true), 1 (somewhat or sometimes true), or 2 (very true or often true). Scores yield raw scores for 8 narrowband scales and 3 broadband scales, which are then transformed into *t* scores based on normative data [28]. The CBCL/6-18 aids in ruling out other pathologies potentially confounding ADHD diagnosis and establishing inclusion criteria for typically developing children. Internal consistencies as measured by Cronbach α for the problem scales range from 0.72 to 0.97 [28].

The CCPT is a computerized test measuring sustained attention and vigilance in individuals aged 6 years and older [41,42]. Performance metrics include measures of reaction times, errors, and response variability. Participants respond to letters displayed on a screen by pressing the spacebar, except when the letter *X* appears. The CCPT-2, chosen as a baseline attention measure, demonstrates good internal consistency (Cronbach α ranges from 0.64 to 0.96) and adequate test-retest reliability (coefficients range from 0.48 to 0.79) [42].

The Brazilian Economic Classification Criterion (CCEB) assesses the socioeconomic status of families based on household properties, educational attainment of the family head, and access to infrastructure [43]. Scores categorize families into socioeconomic levels from A to E. Considering that economically vulnerable children may exhibit more ADHD symptoms and externalizing disorders, this assessment ensures the appropriate pairing of groups to mitigate bias [44].

In addition to standardized instruments, a questionnaire gathered information on the children’s musical experience, preferences, and listening habits at home. Details can be found in Multimedia Appendix 3. At the study’s conclusion, the children were asked about their preferred testing condition.

### Statistical Analysis

The sample size of 46 was determined using G*Power (version 3.010; Universität Düsseldorf), considering α=.05, a medium effect size of 0.25, and a power of 90%. Statistical analyses were conducted using SPSS (version 22.0; IBM Corp) for personal computers. Descriptive statistics were used to calculate the mean and SD for sample characterization. Attention network scores (alerting, orienting, and conflict) for each participant were derived by subtracting error rates, including omissions, perseveration, and outliers, as outlined by Fan et al [40].

**Figure 4:**
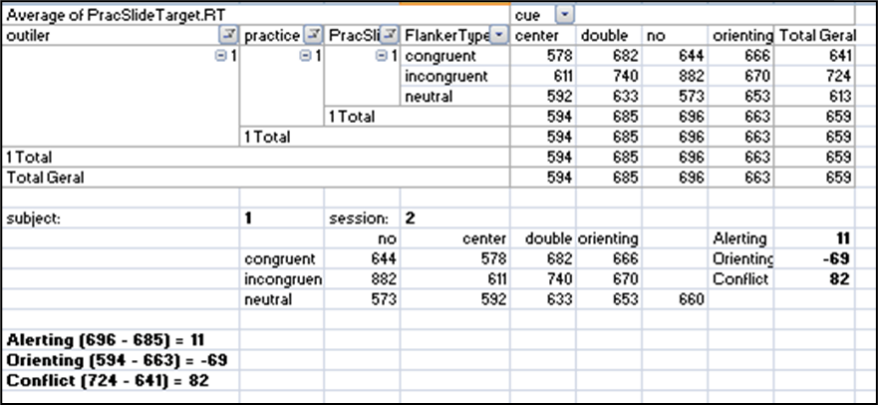
Example of calculations of attentional networks for participant 1, session 2, using an Excel (Microsoft Corp) macro.

**Figure 5:**
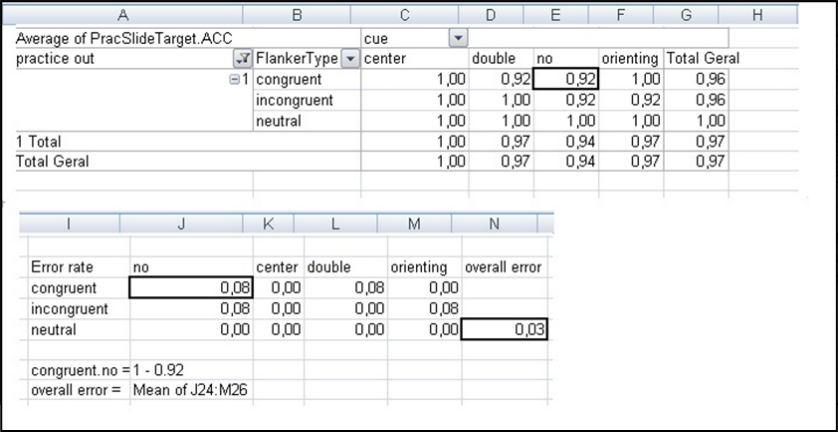
Example of calculations of overall errors of participant 1, session 2, using an Excel (Microsoft Corp) macro. The outlined squares indicate the values used for the calculations in the example.

To compute orienting and alerting scores per participant, the mean reaction time (RT) per cue condition across flanker conditions was calculated (orienting = RT for spatial cue – RT for central cue; alerting = RT for double cue – RT for no cue condition). Conflict scores were obtained by computing the participant’s mean RT for each flanker condition across cue conditions (RT for incongruent – RT for congruent). Mean scores across participants were calculated for each network using an Excel macro obtained from Jin Fan [37]. Error rates were determined by averaging errors across all conditions (cue and flanker). Detailed calculations are illustrated in Figures 4 and 5, based on the Excel macro downloaded from Jin Fan [37].

We used an ANOVA with a repeated measures design to test if the experimental conditions (music×no music), groups (control×ADHD), and their interactions were related to changes in ANT scores. We used 1 model for each attention network measure and error rate. To reduce potential biases arising from individual differences in attention on ANT scores, we included fine-grained age-corrected measures of attention errors from the CCPT. Errors of commission (ie, responding to a stimulus when one should not) and omission (not responding to a stimulus when one should) were entered as covariates in each model.

### Ethical Approval

Ethical approval for this study was obtained from the University’s Ethics Review Committee (97425218.4.0000.5149). Written informed consent was provided by all parents or guardians, while minors provided written informed assent before participation in the trial. The study protocol was initially registered at ReBEC.gov (U1111-12589039). The study adhered to the Transparent Reporting of Evaluations with Non-randomized Designs (TREND) statement [45].

## Results

Descriptive behavioral characteristics of the eligible participants are presented in Table 2. Additional information regarding the participants’ previous musical experiences and emotional connections with the music selections can be found in Multimedia Appendices 1-3. Nearly half of the children reported both familiarity with and enjoyment of the songs used in this study. Moreover, 57 of 76 children (75%) expressed a preference for taking the test while listening to music compared to the no-music condition.

Independent sample *t* tests (2-tailed) revealed no significant age differences between the control and ADHD groups (t_74_=0.47; *P*=.63). Similarly, no significant disparities in socioeconomic status were observed between the groups (t_71_=–1.158; *P*=.25).

**Table 2:** Demographic characteristics of participants (N=76).

Characteristics		TD group (n=42), mean (SD)	ADHD group (n=34), mean (SD)
Age (years)		11.0 (0.85)	10.9 (0.75)
Brazilian Criteria of Economic Classification^a^		28.5 (11.32)	31.5 (11.15)
**SNAP-IV^b^ symptoms (*t* score^c^)**			
	Inattention	1 (1.97)	6 (2.35))
	Hyperactivity/impulsivity	0.5 (0.84)	5 (1.85)
CBCL-ADHD^d^ (*t* score^c^)		44.7 (5.90)	61.6 (11.47)
**CCPT^e^ score**			
	Omissions	11.7 (9.5)	11.9 (8.6)
	Commissions	25.0 (6.0)	25.4 (7.3)

^a^For the Brazilian Criteria of Economic Classification, 0-16=class D and E, 17-22=class C2, 23-28=class C1, 29-37=class B2, 38-44=class B1, and 44-100=class A.

^b^SNAP-IV: Swanson, Noland, and Pelham Scale IV. Each item is categorized as present (1 point, which means all answers are equivalent to 2, “quite a bit,” or 3, “very much”) or absent (0 points, which means all answers equivalent to 0, “not at all,” or 1, “just a little”). The cutoff point for screening ADHD is >5.

^c^Mean *t* scores calculated with reference to Brazilian normative data [25].

^d^CBCL-ADHD: Child Behavior Checklist for Attention Deficit/Hyperactivity Disorder.

^e^CCPT: Conner’s Continuous Performance Test.

### Effect of Music on ANT and Error Rate

Table 3 presents the means and SDs for each ANT measure across different conditions and groups. Repeated measures ANOVA models are shown in Table 4. We did not find significant main effects for music or the interaction between group and music for the alerting, orienting, and conflict attentional networks (all were nonsignificant, with *P* values ranging from .28 to .74). Regarding ANT errors, we found a significant main effect for music with a moderate effect size (*F*_1,72_=9.83; *P*=.03; ηp^2^=0.06) but not for the group×music interaction (*F*_1,72_=1.79; *P*=.18). Both the typically developing participants (mean 0.041, SD 0.036 vs mean 0.039, SD 0.049) and ADHD participants (mean 0.066, SD 0.058 vs mean 0.052, SD 0.042) made fewer errors in the ANT while listening to music.

**Table 3:** Mean reaction time and SDs for correct responses in each condition (music and no music) and in both groups (attention deficit/hyperactivity disorder and typically developing).

Group and flanker condition			Reaction time with music (ms), mean (SD)									Reaction time without music (ms), mean (SD)					
			Central cue		Double cue		No cue		Orienting		Central cue			Double cue		No cue	Orienting
**Typically developing group**																	
	Congruent	639 (120)		626 (266)		692 (130)		619 (192)		635 (135)			614 (177)		675 (229)		612 (296)
	Incongruent	702 (106)		689 (343)		736 (201)		680 (62)		674 (75)			680 (177)		723 (109)		660 (203)
	Neutral	621 (167)		597 (115)		664 (227)		590 (182)		634 (205)			612 (255)		657 (63)		598 (246)
**Attention deficit/hyperactivity disorder group**																	
	Congruent	686 (120)		668 (266)		743 (130)		663 (192)		689 (120)			672 (266)		734 (130)		648 (192)
	Incongruent	763 (106)		722 (343)		796 (201)		693 (62)		759 (106)			708 (343)		781 (201)		710 (62)
	Neutral	672 (167)		658 (115)		732 (227)		647 (182)		651 (167)			648 (115)		709 (227)		629 (182)

**Table 4:** Score comparison for participants with typical development (TD; n=42) and attention deficit/hyperactivity disorder (ADHD; n=34) on the Attentional Network Test with and without music, controlling for Conner’s Continuous Performance Test omission and commission errors (repeated measures ANOVA).

Measure	TD group score, mean (SD)			ADHD group, mean (SD)			Main effect of music			Interaction of music×group	
	No music	Music	No music		Music	*F* test (*df*)		*P* value	*F* test (*df*)		*P* value
								
Alerting	68.62 (52.67)	60.33 (81.28)	79.56 (54.64)		62.32 (64.75)	1.20 (1)		.28	0.15 (1)		.70
Orienting	26.69 (47.6)	24.29 (40.88)	38.62 (50.33)		44.03 (57.8)	0.86 (1)		.36	0.28 (1)		.60
Conflict	58.02 (47.06)	53.81 (38.28)	56.18 (47.35)		56.50 (38.28)	0.11 (1)		.74	0.12 (1)		.73
Error rate^a^	0.041 (0.036)	0.039 (0.049)	0.066 (0.058)		0.052 (0.042)	8.83 (1)		.03	1.79 (1)		.18

^a^ηp^2^=0.06.

## Discussion

### Principal Findings

The results across both ADHD and control groups revealed neither a significant main effect of music in attention networks, as indexed by the ANT, nor a significant interaction between music and group. However, a significant main effect was found in the overall number of errors during the ANT, suggesting listening to music decreases the error rate.

This study hypothesized that listening to music during testing may improve the attention performance of children with ADHD. However, our findings did not fully support this hypothesis. We found listening to music can improve performance accuracy by decreasing the number of errors, and this happened in both groups. The deficits in the attentional networks of children with ADHD assessed through the ANT are still controversial, and there are previous studies that also did not find differences in the efficiency of their networks when compared to children without ADHD [24,46]. Also, higher alertness seems to be associated with increased error rates [45,47,48], so music helped promote an optimal condition, that is, one that would not affect the accuracy of the attentional network, or it generated a weak effect size for the detection of differences.

The effects of music on cognitive performance are affected by motivation, especially if it is a favorite song [48]. In this study, most of the participants reported positive feelings (ie, liking the song and feelings of happiness) about the pieces of music used, and they preferred to perform the ANT listening to music. This may have increased the motivation to complete the task, consequently contributing to making fewer mistakes while listening to music, but this is only speculative. In the case of the children with ADHD, they may have lower levels of motivation and self-regulation problems, which lead to the devaluing of rewards that are not immediate in comparison to typically developing children [49,50]. Although we did not find a significant difference between the groups, our results suggest a tendency for the effect of music to be more significant in the group with ADHD, which corroborates studies on the role of motivation in achieving school tasks [51-53]. Children with ADHD, when motivated, are more likely to try harder when faced with difficulties or not to give up when something is difficult to finish or is not interesting to them [54]. So, it is important to understand that strategies that motivate these children can directly affect their performance of a task, and this does not necessarily have to be through an attentional route.

Also, previous studies demonstrated that when music has lyrics, it might impair performance attention [55,56], which could have been another important factor that contributed to the reduction of errors during the performance of the task in our study. Still, our musical stimulus was composed of songs that could have created an atmosphere to captivate the children, as 3 of the songs were from games that these children routinely played. This may have generated a feeling of reward and motivated the children more during the test.

The concept of affect-matching music refers to the idea that individuals tend to seek out and prefer music that aligns with their current emotional state or desired emotional state. Improvements in cognitive performance are facilitated by listening to affect-matching music [57]. On the other hand, music is also capable of inducing emotions [12,15]. In this study, we chose to use music that was familiar and preferred by most participants, so it is possible that the combination of these factors, music that induces emotions, plus the listener’s perception (emotion and arousal), led to our results.

Since it was developed, the ANT has been widely used by the scientific community in diverse cultures and investigations (for, eg, anxiety, ADHD, bilingualism, borderline personality disorder, deafness, mindfulness training, schizophrenia, and time of day) [46,58], and the child variant [36] is the gold standard in this population, being engaging and visually stimulating. Thus, it was the best tool to assess the effect of music on performance attention.

This study has some limitations. First, the results are only generalizable to the specific music used in this study and potentially to other music in the same genre and with a specific visual task in a laboratory setting. It will be necessary to carry out the same study with the same type of music while performing school tasks or in the classroom.

Second, the sample of children with ADHD was recruited from only one clinical care setting, and this may have generated biases. Also, because it is a single clinical sample, the findings may not generalize to the broader population, limiting the external validity of the study.

### Conclusion

Our findings, while preliminary, suggest that music does not appear to interfere with attentional networks. However, they do indicate that listening to music reduces the number of errors during directed attention tasks such as the ANT. Can similar results be observed during academic tasks? Could listening to music serve as a means to motivate children, thereby enhancing their engagement and accuracy in completing tasks? These questions warrant further investigation.

The motivational significance of a task plays a crucial role in channeling the additional effort required to sustain attention, potentially contributing to the reduction in errors observed. However, the effects of music on attention may vary among individuals with ADHD. While some children may find certain types of music beneficial for enhancing attention, others may find it disruptive. Therefore, it is essential to consider personal preferences and sensitivities when assessing the impact of music on attention in children with ADHD.

Ultimately, our research underscores the importance of exploring alternative and complementary treatments for ADHD that incorporate music, as it possesses intrinsic motivating potential and is readily accessible in people’s daily lives. Further studies are needed to deepen our understanding of how music can be effectively used to support attention and cognitive function in individuals with ADHD.

## Appendix

Multimedia Appendix 1 Assessment of emotional state.

Multimedia Appendix 2 Frequency (percentage) of answers to the questionnaire to assess the emotional relationship between listener and songs.

Multimedia Appendix 3 Frequency (percentage) of answers of preview musical experience questionnaire.

## Abbreviations

ADHD: attention deficit/hyperactivity disorder
ANT: Attention Network Test
CBCL/6-18: Child Behavior Checklist for Ages 6-18
CCEB: Brazilian Economic Classification Criterion
CCPT: Conner’s Continuous Performance Test
K-SADS-PL: Schedule for Affective Disorders and Schizophrenia for School-Age Children–Present and Lifetime
ODD: oppositional defiant disorder
RT: reaction time
SNAP-IV: Swanson, Noland, and Pelham Scale IV
TREND: Transparent Reporting of Evaluations with Non-randomized Designs

## Data Availability

The data analyzed during this study are available from the corresponding author on reasonable request.
