# Supplementary material for: Effects of Background Music on Attentional Networks of Children With and Without Attention Deficit/Hyperactivity Disorder: Case Control Experimental Study
Source: Interact J Med Res. 2024 Jul 18;13:e53869. doi: 10.2196/53869 (PMC11294770; doi:10.2196/53869)
Supplement: Multimedia Appendix 1 [file ijmr_v13i1e53869_app1.docx]

**Multimedia Appendix 1. Assessment of Emotional State**

1. Do you this song?

Yes Maybe No

1. How do you feel when you listen this song?


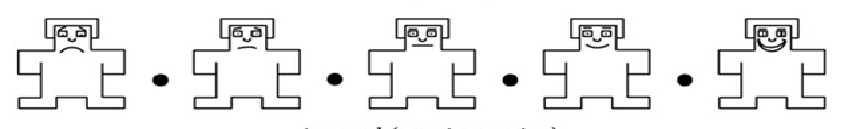


1 2 3 4 5

Valence


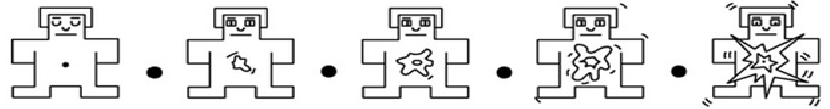


1 2 3 4 5

Arousal

1. Do you like this song?


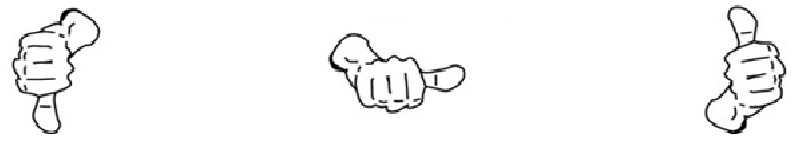


No Neutral Yes
