# Supplementary material for: Effects of Background Music on Attentional Networks of Children With and Without Attention Deficit/Hyperactivity Disorder: Case Control Experimental Study
Source: Interact J Med Res. 2024 Jul 18;13:e53869. doi: 10.2196/53869 (PMC11294770; doi:10.2196/53869)
Supplement: Multimedia Appendix 2 [file ijmr_v13i1e53869_app2.docx]

|  |  | F | (%) |
| --- | --- | --- | --- |
| Do you know this song? |  |  |  |
|  | Yes | 41 | 53 |
|  | Maybe | 14 | 18 |
|  | No | 21 | 5 |
|  |  |  |  |
| Do you like this song? | Don't like | 4 | 5 |
|  | Neutral | 25 | 33 |
|  | Like | 47 | 62 |
|  |  |  |  |
| How do you feel listening this song?^a^ |  |  |  |
| Valence | Very Sad/Sad | 1 | 1.3 |
|  | Neutral | 15 | 9 |
|  | Happy/very happy | 60 | 78 |
| Arousal | Non arousal/Low | 5 | 6.5 |
|  | Neutral | 17 | 22 |
|  | Arousal/very arousal | 54 | 71.5 |

*Note.* We grouped the likert scale rate into 3 categories, 1 and 2 points, 3 points, and 4 and 5 points, for arousal and valence.
