# Supplementary material for: Effects of Background Music on Attentional Networks of Children With and Without Attention Deficit/Hyperactivity Disorder: Case Control Experimental Study
Source: Interact J Med Res. 2024 Jul 18;13:e53869. doi: 10.2196/53869 (PMC11294770; doi:10.2196/53869)
Supplement: Multimedia Appendix 3 [file ijmr_v13i1e53869_app3.docx]

|  |  | F | (%) |
| --- | --- | --- | --- |
| Have you ever taken a music class? |  |  |  |
|  | Yes | 44 | 57 |
|  | No | 32 | 43 |
| How often do you listen to music at home? |  |  |  |
|  | Always | 24 | 31 |
|  | Sometimes | 43 | 56 |
|  | Almost never | 9 | 13 |
|  |  |  |  |
| How often do you listen to music while study? |  |  |  |
|  | Always | 5 | 8 |
|  | Sometimes | 18 | 23 |
|  | Almost never | 53 | 69 |
| What type of music do you listen to the most? |  |  |  |
|  | Funk | 19 | 25 |
|  | Pop (international) | 13 | 17 |
|  | Pop (brazilian music) | 4 | 5.2 |
|  | Hip-hop | 9 | 12 |
|  | Sertanejo | 7 | 9.2 |
|  | Eletronic music | 18 | 23.6 |
|  | Gospel music | 3 | 4 |
|  | Others (gender not identificated, i.e rap of anime, soundtracks of games) | 3 | 4 |
